# Social Cognition Deficits as a Target of Early Intervention for Psychoses: A Systematic Review

**DOI:** 10.3389/fpsyt.2019.00333

**Published:** 2019-05-15

**Authors:** Yuji Yamada, Takuma Inagawa, Kazuki Sueyoshi, Norio Sugawara, Natsuki Ueda, Yoshie Omachi, Naotsugu Hirabayashi, Madoka Matsumoto, Tomiki Sumiyoshi

**Affiliations:** ^1^Department of Psychiatry, National Center Hospital, National Center of Neurology and Psychiatry, Tokyo, Japan; ^2^Department of Preventive Intervention for Psychiatric Disorders, National Institute of Mental Health, National Center of Neurology and Psychiatry, Tokyo, Japan; ^3^Department of Clinical Epidemiology, Translational Medical Center, National Center of Neurology and Psychiatry, Tokyo, Japan

**Keywords:** first-episode psychosis, schizophrenia, ultra-high risk, at risk mental state, theory of mind, emotion recognition, randomized controlled trial

## Abstract

**Backgrounds:** Social cognition deficits are a core feature of schizophrenia and deteriorate functionality of patients. However, evidence is sparse for the treatment effect on social cognition impairments in the early stage of psychosis. Here, we provide a systematic review of the literature on social cognitive impairment in early psychosis in relation to its intervention.

**Methods:** A literature search was conducted on English articles identified by Web of Science and PubMed databases, according to the guidelines of the Preferred Reporting Items for Systematic Reviews and Meta-analyses (PRISMA) statement.

**Results:** Five papers met the inclusion criteria. Results from two studies of cognitive training and one study of modafinil indicate positive results regarding social cognition outcomes in patients with early psychosis. On the other hand, two studies with oxytocin and modafinil did not suggest such effects.

**Conclusions:** Further research is warranted to explore the benefit of early intervention into disturbances of social cognition in psychoses.

## Introduction

Schizophrenia affects approximately 0.7% of the world’s population (1) and is characterized by positive (hallucinations, delusions), negative (apathy, anhedonia, social withdrawal, etc.), and cognitive symptoms. The first signs and symptoms usually appear between the end of adolescence and beginning of early adulthood. The disease has a chronic course with continual psychotic episodes that generally lead to deterioration in cognitive and social functioning (2, 3), as well as unemployment in more than 70% of patients at the chronic stage (4, 5).

Cognitive impairment is a core feature of schizophrenia and is present over the course of the illness (6). Research has shown that neurocognitive domains, such as memory, attention, executive functions, language, and intelligence, are most severely affected (7). Similar impairments are also found in social cognition (8), i.e., mental operations underlying social behavior. Social cognition is understood as a multidimensional construct that comprises emotional processing, social perspective and knowledge, attributional bias, and theory of mind (ToM). Some studies report that social cognition explains the variance of functional outcome more effectively than does neurocognition. Thus, social cognition has been considered an important treatment target for functional improvement in people with psychoses (9–12).

Impairment of social cognition, including emotional recognition (13, 14), ToM (15), and attributional biases (16), is evident before the onset of psychosis, continues throughout the early phase of illness, and may even worsen during the first episode (17–19). There have been attempts to determine the relationship between social cognition and social functioning in early psychosis (20). Available research suggests that deficits in social functioning due to social cognition deficits are present early in the course of psychotic disorders (21–23) and also in first-degree relatives of patients (24, 25).

Individuals in the early phase of psychosis exhibit a greater brain plasticity and milder structural and functional brain changes than those in patients with chronic illnesses, providing the rationale for early treatment (26, 27). So far, most published trials of cognitive remediation have used middle-aged, chronically ill patients (28), and its efficacy for those in the prodromal phase or first episode of psychotic illness is largely unknown. As data from current pharmacological interventions suggest limited effects on social cognition impairments of schizophrenia (29, 30), there is a clear need to develop effective therapeutics to target them.

Here, we provide a systematic review of the literature regarding intervention for social cognition deficits in individuals with early psychosis or high risk for developing psychosis.

## Materials and Methods

### Data Sources and Search Terms

This systematic review was performed based on the PRISMA guidelines (31). From inception to March 15, 2019, YY and TI independently examined the Web of Science and PubMed databases. The following search terms were used as keywords: (“early psychosis” OR “first-episode psychosis” OR “FEP” OR “first-episode schizophrenia” OR “ultra-high risk” OR “UHR” OR “psychosis prodrome” OR “at risk mental state” OR “ARMS” OR “clinical high risk”) AND (“social cognition” OR “theory of mind” OR “emotion recognition” OR “attributional style” OR “social knowledge” OR “social perception”) AND (“training” OR “rehabilitation” OR “remediation” OR “cognitive behavioral therapy” OR “CBT” OR “intervention” OR “pharma*” OR “drug” OR “antipsychotics” OR “antidepressant”) AND (“randomized controlled trial” OR “RCT”). Only studies with human participants and written in English were included. The senior reviewer (TS) approved the final list of the studies included.

### Eligibility Criteria

Prespecified inclusion criteria were as follows: 1) randomized controlled trials (RCTs) comparing a social cognition intervention with treatment as usual, a minimal educational intervention, sham training, or placebo therapy; 2) participants were adults or adolescents between 10 and 40 years old diagnosed with early psychosis (i.e., schizophreniform disorder, schizophrenia, or schizoaffective disorder) (<5 years illness duration) without a) current substance dependence on alcohol or drugs, b) intellectual disability (intelligence quotient <70), c) a history of a significant neurological disorder, and d) florid psychotic or related symptoms likely to require immediate intervention (e.g., suicidality); 3) interventions were training or pharmacotherapy targeted to one or more social cognition domains; 4) comparisons were treatment as usual, a minimal educational intervention, sham training, or placebo therapy; and 5) outcomes were objective scales defined as ToM, emotion recognition, attributional style, social perception, and social knowledge.

#### Outcome Measures

Outcome measures identified by this search were discussed in relation to three domains of social cognition, i.e., emotion recognition, theory of mind (ToM), and attributional bias (see **Table 1**).

**Table 1:** Cognitive scales used.

Study (year)	Emotion recognition	Theory of mind	Attributional bias
Scoriels et al. (2011) (32)	ERT	−	−
Lees et al. (2017) (33)	MCCB-social cognition	−	−
Cacciotti-Saija et al. (2015) (34)	FEEST Movie Stills Task	FBPST, Faux Pas Task, Empathy Quotient, RMET	Ambiguous Intentions Hostility Question
Fernandez-Gonzalo et al. (2015) (35)	POFA	ToM 1st order, ToM 2nd order, Hinting Task, RMET	IPSAQ
Mendella et al. (2015) (36)	MSCEIT	−	−

ERT, Emotion Recognition Task; MCCB,Measurement and Treatment Research to Improve Cognition in Schizophrenia (MATRICS) Consensus Cognitive Battery; FEEST, Facial Expressions of Emotions Task; POFA, Pictures of Facial Affect; MSCEIT, Mayer–Salovey–Caruso Emotional Intelligence Test; FBPST, False Belief Picture Sequencing Task; RMET, Reading the Mind in the Eyes Test; IPSAQ, Internal, Personal and Situational Attributions Questionnaire.

#### Emotion Recognition

Mayer–Salovey–Caruso Emotional Intelligence Test (MSCEIT) (37) measures the participant’s ability to perceive, use, understand, and regulate emotions, while Facial Expressions of Emotions Task (FEEST) (38) requires subjects to identify six basic emotions (happiness, sadness, anger, fear, surprise, and disgust) from facial expressions, although Cacciotti-Saija et al. (34) gave no information about whether they used morphing images of different emotional valences or varying degree of emotional intensities. Movie Stills Task (39) requires identification of emotions (happy, surprised, afraid, angry, disgusted, sad, or neutral) from a complex movie scene. On the other hand, Pictures of Facial Affect (POFA) (40) uses facial photos providing the morphing faces of different emotions, or emotional face of different emotional intensities (0% fearful, 10% fearful, 20% fearful, 30% fearful, … and 100% fearful). In this task, subjects are instructed to recognize basic emotions (happiness, sadness, anger, disgust, and surprise) in 60 faces. Furthermore, Emotion Recognition Task (ERT) consists of a series of mixed ethnic background faces photographs depicting seven emotions: happiness, surprise, neutral, fear, disgust, anger, and sadness (41). Finally, a subdomain of social cognition of the MATRICS Consensus Cognitive Battery (MCCB) (42, 43) was developed for use in schizophrenia.

#### Theory of Mind

False Belief Picture Sequencing Task (44) consists of arrangement of picture-cards into a logical sequence of events to test the ability to go beyond objective information to reason that a story protagonist is acting on the basis of a false belief. Reading the Mind in the Eyes Test (RMET) (45) assesses the ability to infer mental states from images of eye regions, and provides a sensitive measure of social cognition impairments in early psychosis (46). The modified Faux Pas Task (47) requires participants to respond when faux pas are present. The Empathy Quotient (48) is a self-report measure of cognitive and affective aspects of empathy.

ToM task consists of four classic false belief/deception stories; the “Sally & Anne” (49) and “Box of Chocolate” stories (50) are used to assess first-order ToM abilities, while “Burglar” (50) and “Ice-Cream Van” (50) are used to assess second-order ToM skills. These stories are read aloud by the examiner, and subjects are asked to listen and subsequently answer a ToM question and a control question. In order to avoid a possible learning effect, two homologous false belief/deception first-order ToM stories [“Cigarettes” (51) and “Piggy bank” (51)] and second-order ToM stories [“Train station” (52) and “Coke” (52)] are administered at baseline and posttreatment. Hinting Task (53) is also used, in which patients have to understand indirect speech and infer the mental state of one of the characters.

#### Attributional Bias

Ambiguous Intentions Hostility Questionnaire (54) contains five short vignettes describing negative interpersonal events with ambiguous causality. Internal, Personal, and Situational Attributions Questionnaire (IPSAQ) (55) is designed to assess the extent to which individuals attribute negative and positive events to different attributional loci. The task consists of 32 social items describing 16 positive and 16 negative events. Patients are asked to generate the most likely cause of each event and state whether the cause is due to self, other people, or circumstances. Six subscale scores are generated (number of positive events attributed to self, other people, and circumstances, and corresponding scores for negative events), which are used to calculate two composite scores: externalizing bias (EB) and personalizing bias (PB).

### Procedures and Data Extraction

Initially, titles and abstracts were screened to identify eligible studies. Full-text articles were obtained for all the studies considered compatible based on the abstract screening and were further reviewed for eligibility.

### Risk of Bias in Individual Studies

We selected the Cochrane Collaboration’s risk of bias tool to evaluate risk of bias in each trial. Two independent reviewers (YY and TI) determined 1) if patients were correctly randomized, 2) if the randomization method was properly concealed, and 3) if subjects and/or investigators and/or raters were blinded. We assessed whether the authors collected and reported all results for all pre-specified outcomes. A senior reviewer (TS) approved the final decision of the assessment of risk of bias.

## Results

**Figure 1:**
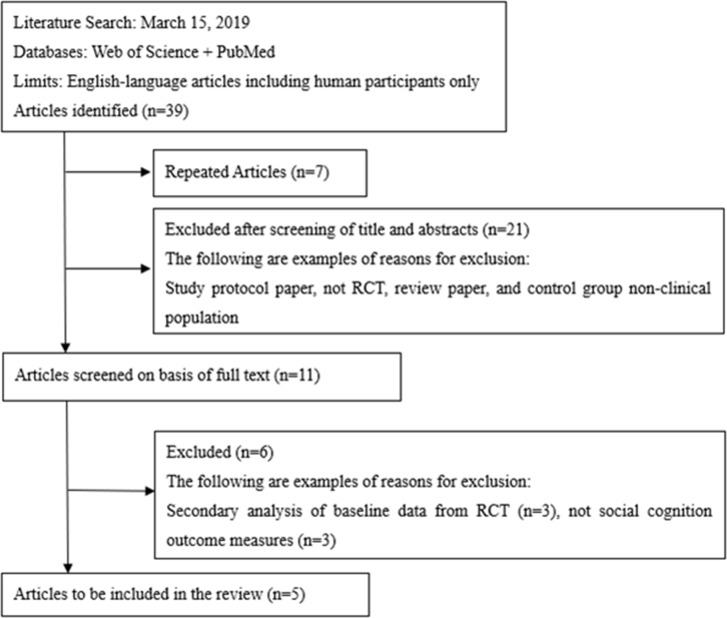
Study selection flowchart, following the guidelines of the PRISMA statement. Initially, titles and abstracts were screened to identify eligible studies. Full-text articles were obtained for all the studies considered compatible, based on the abstract screening, and were further reviewed for eligibility.

**Figure 2:**
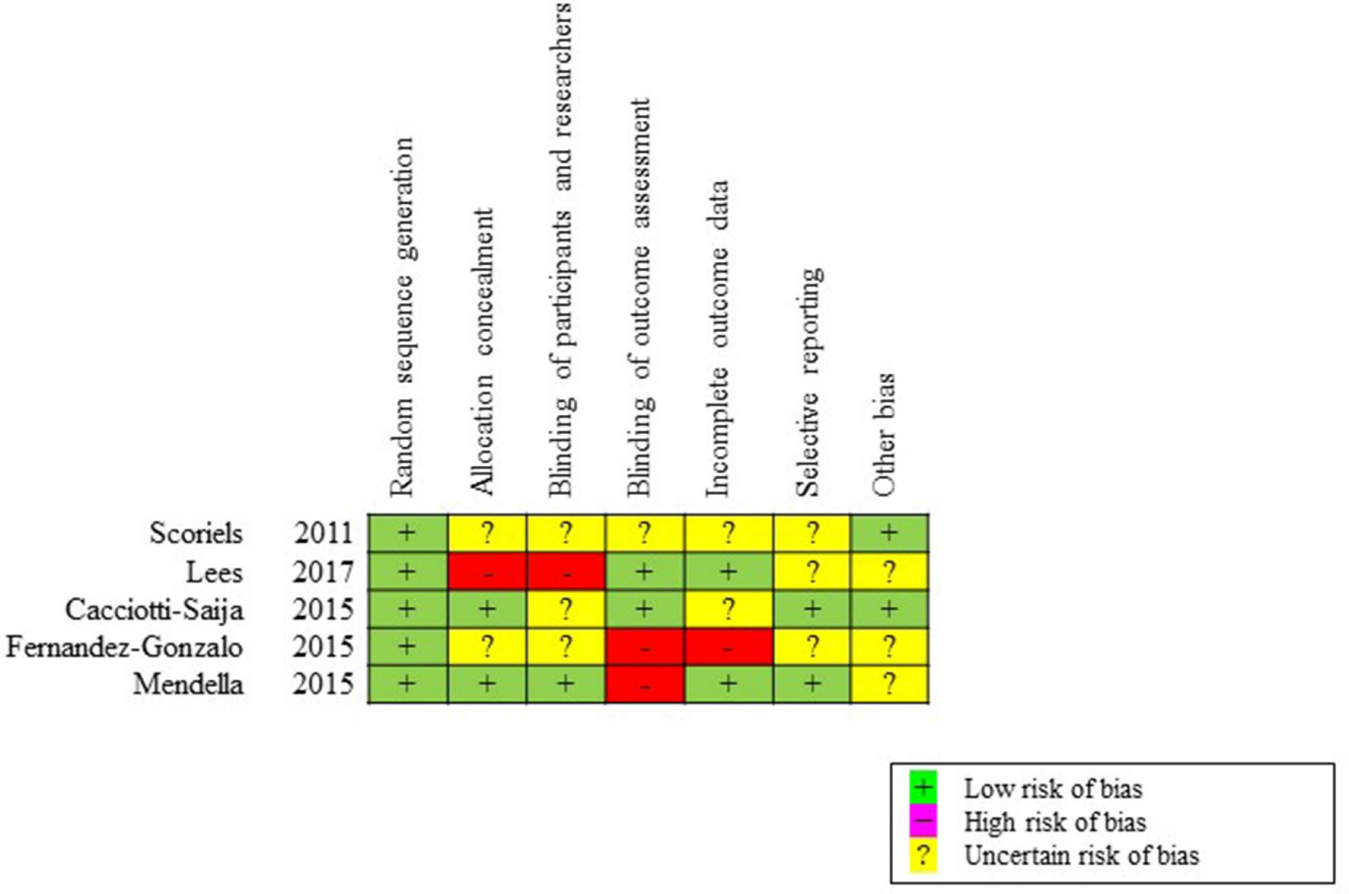
Assessment of risk of bias for included studies, based on the Cochrane Collaboration’s risk of bias tool. We determined whether each trial had a low, high, or uncertain risk of bias in terms of random sequence generation, allocation concealment, blinding of participants and personnel, blinding of outcome assessment, incomplete outcome data, selective reporting, and other biases.

The initial search provided a total of 39 records. After removing duplicates, 32 articles were screened, of which 11 English full texts were available. Five articles found eligible for the systematic review. Articles describing studies that involved only secondary analysis of baseline data from RCT (*n* = 3), and no social cognition outcome measures (*n* = 3) were excluded. The PRISMA study selection flowchart is shown in **Figure 1**. The summary of risk of bias is presented in **Figure 2**.

### Characteristics of Studies

The five studies included in the current review encompassed 212 subjects (151 men and 61 women). Characteristics of the selected studies are shown in **Table 2**. There were considerable differences between the studies in terms of demographics, intervention type, and outcome measures. Two studies (32, 36) targeted first-episode psychosis (FEP) subjects, while three (33–35) included early psychosis patients with less than 5-year illness duration. Two studies used cognition training or rehabilitation as their intervention (35, 36), while one concerned intervention with oxytocin (34), and two with modafinil (32, 33). For these studies, treatment as usual (35, 36) or placebo therapy (32–34) was used as a comparison group.

**Table 2:** Summary of studies comparing the performance on social cognition tasks in individuals with early psychosis.

Study (year)	Participants (number)	Age (years) Mean (SD)	Gender Male, %	Intervention (number)	Control (number)	Outcome domains	Results
Scoriels et al. (2011) (32)	FEP (40)	Crossover design 25 (2)	77.5	Modafinil (40)	Placebo (40)	Emotion recognition	Significant effects
Lees et al. (2017) (33)	Early psychosis (40)	Crossover design 25.7 (4.9)	73.8	Modafinil (40)	Placebo (40)	Emotion recognition	No significant effects
Cacciotti-Saija et al. (2015) (34)	Early psychosis (52)	Intervention 21.5 (4.2) Control 22.3 (4.4)	69.2	SCT + Oxytocin (27)	SCT + Placebo (25)	Emotion recognition Theory of mind Attributional bias	No significant effects in any domains
Fernandez-Gonzalo et al. (2015) (35)	Early psychosis (53)	Intervention 30.9 (5.9) Control 30.0 (7.4)	64.2	NPT-MH (28)	Nonspecific computer training (25)	Emotion recognition Theory of mind Attributional bias	Significant effects only in emotion recognition
Mendella et al. (2015) (36)	FEP (27)	Intervention 25.0 (3.9) Control 24.8 (2.6)	74.1	CCT (16)	TAU (11)	Emotion recognition	Significant effects

FEP, first-episode psychosis; SD, standard deviation; SCT, social cognition training. Participants underwent a 6-week group-based program. The program involved a combination of group learning activities (70% of total session time) and computer-based training tasks (30% of session time) completed in pairs. NPT-MH, Neuro Personal Trainer–Mental Health; a new cognitive exercise based on multimedia content; CCT, compensatory cognitive training; TAU, treatment as usual.

### Systematic Review

#### Social Cognitive Deficits at Baseline

Social cognitive impairment, including emotional recognition, ToM, and attributional biases, was evident during the early phase of psychosis, as shown in **Table 3**.

**Table 3:** Effect of intervention on social cognition performance.

Study	Scales	Intervention		Control		p-value	Effect size
		Baseline mean (SD) score	Posttreatment mean (SD) score	Baseline mean (SD) score	Posttreatment mean (SD) score		Partial η^2^
Scoriels et al. (32)	ERT-sadness	83.6 (3.18)	91.8 (2.09)	83.6 (3.18)	91.8 (2.09)	0.003	0.330 (Hedges’ *g*)
Lees et al. (33)	MCCB-social cognition	38.8 (9.4)	40.2 (11.5)	38.8 (9.4)	40.2 (11.5)	0.22	0.139 (Hedges’ *g*)
Cacciotti-Saija et al. (34)	FEEST Movie Stills—no face Movie Stills—face FBPST Faux Pas—Hit Rate Faux Pas—False Alarm Empathy Quotient RMET AIHQ—Hostility Bias AIHQ—Blame AIHQ—Aggression	45.0 (7.3) 9.8 (1.8) 11.9 (3.0) 18.7 (4.7) 0.9 (0.1) 0.2 (0.3) 11.8 (6.2) 66.1 (17.0) 26.2 (10.3) 42.4 (14.9) 22.8 (7.3)	49.4 (6.5) 10.3 (2.2) 11.7 (2.0) 21.1 (4.8) 0.9 (0.2) 0.1 (0.2) 10.9 (4.8) 66.6 (17.8) 23.2 (8.4) 41.8 (14.3) 22.6 (9.3)	44.9 (7.5) 9.7 (1.8) 11.3 (1.6) 20.1 (4.8) 0.8 (0.2) 0.1 (0.3) 12.6 (4.7) 69.7 (14.2) 21.3 (3.6) 41.1 (12.4) 22.3 (4.4)	48.8 (8.3) 10.3 (2.1) 11.7 (1.7) 20.7 (5.2) 0.9 (0.2) 0.1 (0.2) 13.0 (4.8) 71.7 (14.4) 19.1 (3.3) 38.8 (11.3) 21.0 (4.0)	0.93 0.88 0.44 0.12 0.09 0.73 0.21 0.53 0.67 0.36 0.79	0.001 0.002 0.015 0.042 0.047 0.006 0.032 0.013 0.007 0.020 0.004
Fernandez-Gonzalo et al. (35)	POFA ToM 1st order ToM 2nd order Hinting Task RMET IPSAQ—Externalizing IPSAQ—Personalizing	45.6 (6.0) 3.9 (0.5) 3.1 (0.8) 4.6 (1.3) 23.1 (4.5) 0.2 (3.0) 1.1 (0.6)	50.2 (5.0) 3.9 (0.3) 3.1 (1.1) 5.6 (0.8) 24.1 (5.2) 3.6 (14.5) 1.0 (0.8)	45.2 (5.0) 3.8 (0.6) 3.1 (1.0) 4.2 (1.4) 22.4 (4.7) 0.0 (3.4) 1.2 (0.5)	46.8 (4.2) 3.9 (0.3) 2.6 (0.8) 5.5 (0.9) 22.0 (4.9) −0.1 (2.8) 1.1 (0.4)	0.009 0.76 0.25 0.53 0.25 0.32 0.98	0.167 0.003 0.035 0.011 0.035 0.027 <0.001
Mendella et al. (36)	MSCEIT	42.8 (12.2)	47.3 (9.5)	46.3 (10.8)	42.3 (10.7)	0.04	0.17

SD, standard deviation, Effect sizes (partial η^2^) indicate small > 0.01, medium > 0.06, and large > 0.14 effects. ERT, Emotion Recognition Task; MCCB, MATRICS Consensus Cognitive Battery; FEEST, Facial Expressions of Emotions Task; FBPST, False Belief Picture Sequencing Task; RMET, Reading the Mind in the Eyes Test; AIHQ, Ambiguous Intentions Hostility Question; POFA, Pictures of Facial Affect; IPSAQ, Internal, Personal and Situational Attributions Questionnaire; MSCEIT, Mayer–Salovey–Caruso Emotional Intelligence Test.

#### Effect Sizes of Interventions for Social Cognitive Deficits

##### Social Cognitive Training

Social cognitive training exhibited significant effects in limited domains. The three studies included in the systematic review used social cognitive training, two of which found significant effects, as shown in **Table 2**. Effect sizes by means of Cohen’s *d* (56, 57) indicated large effects on emotional recognition domains in two studies (35, 36), while other domains were not affected (see **Table 3**).

##### Pharmacological Treatment

There were no significant effects of oxytocin on any outcomes of social cognition (34). One study found that modafinil significantly improved the recognition of sad facial expressions (32), although there was no significant effect on social cognition performance, as measured by the MCCB, in another study of modafinil (33) (**Table 3**).

## Discussion

Five papers met the inclusion criteria for the current review. Two studies of cognitive training showed positive results in terms of social cognition. One study (32) of modafinil also reports improvement of recognition of sad facial expressions. On the other hand, two pharmacological studies (33, 34) on oxytocin or modafinil did not exhibit such effects.

Social cognition training was shown to improve emotional processing in early psychosis (35). Patients with first-episode schizophrenia present difficulties in identifying facial emotions, specifically negative ones (58), which have been related with functionality (9). Current reviews suggest that emotional processing may be improved by cognitive training even at early stages of the illness. On the other hand, efficacy of cognitive remediation was not evident in other domains of social cognition, which requires further investigations.

Social cognitive training programs aim to improve specific domains of social cognitive impairments that are related to social functioning and readily transferable to real-world situations (59). These cognitive models of early psychosis rest on aberrant salience and biased appraisal processes (60). These biological processes consist of increased striatal dopamine release, which is associated with aberrant salience. Aberrant salience opens the gates to consciousness for trivial stimuli to enter the center of attention, and the salient stimulus cries out for an appraisal (60, 61). The appraisal process elicited by aberrant salience is a key mechanism of developing delusions. A characteristic of individuals with early psychosis is that they are still open for multiple explanations for extraordinary experiences. Cognitive therapy targets appraisal processes that accompany perceptual aberrations and suspiciousness to normalize extraordinary experiences with education (61).

Although Cacciotti-Saija et al. (34) and Fernandez-Gonzalo et al. (35) used the same Ekman’s photos as dependent measure, their studies reported different intervention effects. This suggests that social cognitive training and oxytocin treatment may change different neurobiological substrates.

Although existing evidence indicates that oxytocin impacts favorably on domains of social cognition (62), its treatment effects, in comparison with placebo, were absent in young people with early psychosis. Oxytocin is a neuropeptide that interacts with a variety of neuromodulators, including serotonin and dopamine, in the nucleus accumbens and amygdala (63). Results from a previous study (64) suggest that genetic variants of oxytocin receptors may be responsible for social cognitive impairments of schizophrenia. The reliability of benefits of oxytocin and other neuropeptides, e.g., vasopressin, across population and contexts remains an ongoing issue.

Modafinil is a wake-promoting agent for the treatment of excessive daytime sleepiness. It activates monoamines and glutamate, and inhibits γ-aminobutyric acid neurotransmitters in several brain regions, including the prefrontal cortex, hippocampus, hypothalamus, thalamus, and basal ganglia. Modafinil also induces changes of neurotransmissions in the hippocampus and limbic regions, an action related to memory- and mood-enhancing properties (32).

Scoriels et al. (32) reported the efficacy of modafinil on emotional recognition using the Emotion Recognition Task (ERT). Critical nodes in the emotional face recognition circuitry include the amygdala, which is activated during performance on the ERT (65). Modafinil activates amygdala (66) and increases serotonin levels in it (67). These observations suggest that modafinil improves emotional face recognition in patients with FEP through serotonergic effects on the amygdala. On the other hand, Lees et al. (33) did not find the ability of modafinil to improve social cognition in early psychosis, as measured by the MCCB. These results indicate that the prosocial cognition effects of modafinil or other compounds depend on the type of cognitive tests used.

The neural network of social cognition consists of orbitofrontal cortex, medial prefrontal cortex, superior temporal sulcus, and amygdala, whose functional connectivity is decreased in psychotic patients (68, 69). Previous studies showed that the amygdala plays a key role in perception of facial emotional expression (39), while the prefrontal cortices are strongly associated with ToM (70). On the other hand, the superior temporal sulcus is related to both domains of social cognition (71). These lines of evidence may provide a clue to the development of novel therapeutics, including those of neuromodulation methods.

The differential effects of treatment on emotion recognition, ToM, and attribution styles deserve discussions. Emotion processing shows a consistent relationship with community functioning, which includes a wide range of activities and behaviors related to work functioning and independent living (72, 73). ToM relates to the capacity to interpret beliefs and feelings of others, i.e., predicting general psychotic symptoms, especially negative ones (74). Moreover, ToM is strongly associated with multiple dimensions of social functioning, including interpersonal communication, recreational activities, independence, and performance (73). On the other hand, attributional bias describes how individuals make sense the causes of the positive and negative social events and interactions encountered in life, providing a significant impact on behaviors (75). These findings support the roles for individual domains of social cognition in mediating neurocognition and functional outcomes, which may be relevant to early psychosis.

To conquer social cognition impairments in established schizophrenia, psychosocial approaches, e.g., social cognition and interaction training (SCIT), metacognitive training, training of affect recognition (TAR), emotion and ToM imitation training, emotion processing, and ToM video-based training, as well as pharmacological approaches, e.g., aripiprazole and risperidone, have been attempted. However, there is no such attempt targeting early psychosis, indicating a need for further efforts in this area.

Since no definite strategy has been established to treat social cognition deficits in early psychoses, some types of neuromodulation have been drawing attention. For example, repetitive transcranial magnetic stimulation (rTMS) has been shown to ameliorate facial affect recognition, assessed by “Picture of Facial Affect,” in patients with chronic schizophrenia (76). This result may indicate that noninvasive brain stimulations may improve social cognition in patients with psychosis. Transcranial direct current stimulation (tDCS) is another type of transcranial electrical stimulation procedures. So far, tDCS has been shown to improve neurocognition, as well as daily-living skills and depressive symptoms, in patients with schizophrenia (77). Of note, the effect on psychotic symptoms was associated with oxy-hemoglobin concentrations in cortical regions, as measured by near-infrared spectroscopy (78). Based on these considerations, efforts to evaluate the benefit of neuromodulation on social cognition in psychosis are warranted.

In the present review, we did not find any study exploring the influence of antipsychotic treatments on social cognitions, such as ToM, emotion recognition, and attributional style, in patients with early psychosis. This area also deserves further investigations.

## Limitations

The limitations of the present review should be noted here. Although 2006 workshop sponsored by the National Institute of Mental Health (NIMH) (11) recommended five domains (attributional style, emotion recognition, social knowledge, social perception, and ToM) for the evaluation of social cognition in psychotic disorders, no study to date has comprehensively examined these domains in the same sample; heterogeneity in terms of social cognitive domains across studies may have obscured findings on the efficacy of treatments. Further investigations circumventing these methodological issues deserve considerations.

## Conclusions

As interventions into disturbances of social cognition in early psychosis provide an important issue, further studies, including those with novel paradigms, are warranted.

## Author Contributions

YY and TS planned the study. YY designed it and drafted the first manuscript. YY and TI independently searched and assessed the literature. TS approved the final list of included studies. TI, MM, KS, NS, NU, YO, NH, and TS critically reviewed the draft and revised it. All authors made substantial contributions and approved the final manuscript.

## Funding

This study was supported by Japan Society for the Promotion of Science (JSPS) KAKENHI No. 17K10321, Intramural Research Grant (29-1, 30-1, 30-8) for Neurological and Psychiatric Disorders of National Center of Neurology and Psychiatry (NCNP), and AMED under Grant Numbers 18dk0307069 and 18dk0307081.

## Conflict of Interest Statement

The authors declared that the research was conducted in the absence of any commercial or financial relationships that could be construed as a potential conflict of interest.
